# Utilises Machine Learning Techniques to Deeply Analyse the Role of Lysosome‐Dependent Cell Death in Endometrial Cancer and Its Interactions With the Tumour Microenvironment

**DOI:** 10.1111/jcmm.70939

**Published:** 2025-12-08

**Authors:** Wu Min, Fang Mo, Tang Yun, Yu Guangyu

**Affiliations:** ^1^ Nanxishan Hospital of Guangxi Zhuang Autonomous Region (The Second People's Hospital of Guangxi Zhuang Autonomous Region) Guilin Guangxi China; ^2^ Fuzhou Institute of Technology Fuzhou Fujian China; ^3^ Department of Gynecology Nanxishan Hospital of Guangxi Zhuang Autonomous Region (The Second People's Hospital of Guangxi Zhuang Autonomous Region) Guilin Guangxi China

**Keywords:** endometrial cancer, lysosome‐dependent cell death, machine learning, prognosis prediction, tumour microenvironment

## Abstract

By integrating gene expression data, clinical features and multimodal data, we constructed a machine learning model capable of accurately predicting the prognosis of endometrial cancer patients. The study found that key genes related to lysosome‐dependent cell death exhibit significant expression pattern heterogeneity in endometrial cancer and are closely associated with immune cell infiltration and metabolic characteristics within the tumour microenvironment. Patients in the high‐risk group tend to have lower immune scores and a higher prevalence of immunosuppressive cell types, such as regulatory T cells and M2 macrophages, which may be linked to poorer prognosis and resistance to immunotherapy. Additionally, we discovered that the expression of lysosome‐dependent cell death‐related genes correlates with patients' sensitivity to chemotherapeutic drugs, providing new perspectives for personalised treatment of endometrial cancer. Through this study, we characterised the prognostic relevance of lysosome‐dependent cell death–related genes in endometrial cancer, and identified biomarkers with potential utility for risk assessment and therapeutic stratification.

## 1 Introduction

Endometrial cancer, which in 2020 was one of the most prevalent gynaecological malignancies, is characterised by its heterogeneous nature. Despite recent progress in surgical and chemotherapeutic interventions, this heterogeneity often results in diverse clinical outcomes and treatment responses [1, 2, 3]. However, appreciating the molecular basis of this heterogeneity is important in developing patient‐specific therapeutic regimens and predicting clinical outcomes.

Lysosome‐dependent cell death (LDCD) has recently been identified as an important modality in cancer biology that can affect both tumour progression and therapy response. Loss of lysosomal integrity with consequent leakage of lysosomal enzymes into the cytosol results in cellular self‐digestion and death, and this process is referred to as LDCD [4, 5, 6]. This pathway is at the same time a cell death machinery as well as a modulator of the tumour microenvironment, especially the infiltration of immune cells and metabolic reprogramming.

In this work, machine learning algorithms are implemented to examine the impact of LDCD on endometrial cancer and the interactions with tumour microenvironment. The present study intends to build a powerful patient prognosis prediction model using gene expression data together with clinical features or multisource multimodal datasets [7, 8, 9]. We hereby demonstrate a marked heterogeneity of expression of canonical LDCD genes and their association with the immune cell infiltrating profile and metabolic landscape of the tumour microenvironment.

In addition, we reveal that patients in the high‐risk subgroup with lower immune scores have more immunosuppressive cell types, including regulatory T cells and M2 macrophages, and are associated with unfavourable clinical outcomes and immunotherapy resistance. Moreover, we found that the expression levels of genes involved in LDCD were associated with the sensitivity of the patients to chemotherapeutic drugs, indicating potential implications rather than direct mechanistic inference for endometrial cancer.

We further explore potential biomarkers for prognosis assessment and treatment response prediction in terms of the mechanism of action affecting LDCD in endometrial cancer. These results lay a groundwork for designing more precise and personalised treatments based on both the driver genes and the surrounding environment of the cancer cell, which could ultimately improve outcomes for women with endometrial cancer.

## 2 Methods

### 2.1 Data Source

RNA‐sequencing profiles and corresponding clinical information for uterine corpus endometrial carcinoma (UCEC) patients were obtained from the TCGA‐UCEC cohort. When applicable, GEO endometrial cancer datasets were included as external validation cohorts. For single‐cell transcriptomic validation, we used the GSE139555 dataset, which contains scRNA‐seq data from treatment‐naïve endometrial cancer tissues. All datasets used in this study are exclusively derived from endometrial cancer patients. Any previous mention of lung adenocarcinoma represents a typographical error and has been corrected accordingly [10, 11].

### 2.2 Selection of Core Hub Genes

Identifying hub genes. The core hub genes were first identified by determining the intersection of the predefined lysosome‐dependent cell death (LDCD)–related gene set, and the Venn diagrams were used to visualise the common genes. This filtering served as a first step of candidate elimination for further examination. Next, we performed a protein–protein interaction (PPI) network analysis with the STRING database [12, 13, 14]. Such analyses enabled us to investigate the interaction and connection among the overlapping genes, and to gain insights into the potential roles of the genes in disease mechanisms. Next, the PPI results were imported to Cytoscape, which is an efficient software for network visualisation. Where six LDCD‐related hub genes with stronger connectivity were identified. These hub genes represent centrally positioned LDCD‐associated candidates rather than confirmed pathogenic drivers. In order to explore the potential functions and pathways these core hub genes were involved in, we then conducted a functional enrichment analysis with Metascape. This analysis presented a systematic summary regarding biological processes, molecular functions and pathways these genes participated in, yielding significant clues for the potential roles of these genes in disease development and treatment targets.

### 2.3 Establishment of the Model

Based on the patient's life status and life span, we explored prognosis‐related DEGs. This analysis then indicated potential genes with a profound effect on patient prognosis. A clustering approach identified subtypes of patients to aid in our understanding. This enabled us to discover inherent patterns in the data, which may correspond to distinct prognostic profiles or abnormalities in the disease. After clustering, we created a scoring model to measure the prognostic effect of the gene expression patterns we found. To achieve this, we used PCA on the first two principal components. These components contain most of the variance in the data, thereby ensuring the reliability of the scoring method. This scoring schema allows patients to be stratified on the basis of their gene expression profiles and provides a quantitative number that can be further used to predict survival. By combining these analyses, we hope to enable better prognostic predictions and perhaps allow for individualised treatment options.

### 2.4 Clinical Functional Assessment

We performed systematic analyses to investigate relationships between the model scores and the clinical characteristics of individual patients, including gender, age, stage, pathology and survival status. This included statistical tests to explore the relationship between both factors and how they could affect the model score and vice versa. We investigated the association of model scores with survival in multiple independent clinical features. The Kaplan–Meier survival curves were used to illustrate the different survival probabilities between diverse patient groups classified based on model scores. Log‐rank tests were performed for analysis of the statistical significance of these differences [15, 16, 17]. This study aimed to further assess the influence of individual variables (including model scores) on the survival rate. The prognostic value of each variable was analysed independently. For the analysis of risk factors, we further performed multivariate analysis that took all variables into account at the same time point. This procedure was helpful to determine what factors were predictive of survival when all factors were mutually adjusted for and to give an overall sense of the predictive capabilities inherent in the model.

### 2.5 Immune Infiltration Analysis

The relative percentages of 22 immune cell subgroups in each sample were determined using the CIBERSORT algorithm with a gene expression matrix along with the LM22 signature matrix. The calculation was carried out using 1000 permutations to get a more robust result. Although QN is useful for microarray data, it may not be equally beneficial for RNA‐Seq data, as the distribution of the input expression values is not as skewed. For robust deconvolution, only CIBERSORT *p* values < 0.05 samples were selected for further analysis. Moreover, immune‐related scores such as ImmuneScore, StromalScore and ESTIMATEScore were computed based on the “estimate” R package. These scores signify the degree of immune‐ and stroma infiltration within the tumour microenvironment (TME), which were applied for comparisons of the immune landscapes among patient subgroups [18, 19, 20].

### 2.6 Machine Learning

Data were split into a training set (Dataset A), two independent test sets (Datasets B and C) and an internal validation set (Dataset D), by ensuring a balanced 1:1:1 ratio. There was a strategically balanced representation across cohorts, leading to a particularly strong model for training and testing. We employed a battery of 15 machine‐learning algorithms to thoroughly evaluate the performance of prognostic prediction. These algorithms were chosen for their complementary modeling approaches and demonstrated applicability for survival analysis and high‐dimensional data in biomedicine. In particular, we covered the following types of algorithms:Linear models (e.g., Lasso‐Cox, Elastic Net): because of their ease of interpretation and of being regularised in high dimensions. Tree‐based ensemble methods (e.g., Random Forest, Gradient Boosting, CoxBoost): since they can model complex creativity and high‐order feature interactions. Kernel‐based classifiers, such as SVM for their ability to deal with complex decision boundaries and high‐dimensional input space. Neural networks—due to their ability to learn deep, nonlinear structures from omics data (for their capacity to learn deep, nonlinear structures from omics data). This variation was to maintain a balanced and systematic investigation of model performance across methodological ideologies, thus increasing the robustness and generalisability of the prognostic modeling workflow. All the models were trained on Dataset A. Hyperparameters were searched through a fivefold cross‐validation with the type of hyperparameter tuning being grid search of important parameters (e.g., the regularisation strength of Lasso, the number of trees and depth of Random Forest, the learning rate of XGBoost). In each experiment, early stopping was used if necessary to avoid overfitting. The performance of each model was assessed using external test data (Datasets B and C) as well as internal validation data (Dataset D) based on various metrics, such as the C‐index, AUC, recall and F1‐score. These summary statistics accounted for the discriminative performance, sensitivity and positive predictive value of each model. The models were then ranked by an average of these metrics and the top‐ranked models were chosen for further analysis and clinical interpretability. The internal validation group (Dataset D) acted as a second level of validation to confirm the robustness and generalisability of the model, and to guarantee the robustness of the model's performance with respect to the training cohort. This stepwise model‐building process helped to ensure a robust, credible prognostic model for endometrial cancer [21, 22].

### 2.7 Single‐Cell Level Validation

We utilised single‐cell RNA sequencing data from the GSE139555 dataset, which includes samples from 14 treatment‐naïve endometrial cancer patients. Among the 32 available samples, we selected 28 samples for downstream analysis, including 14 tumour tissues and 14 matched normal adjacent tissues (NAT). Sequencing was performed using the Illumina HiSeq 4000 platform. After applying stringent quality control criteria—excluding cells with fewer than 200 detected genes or more than 20% mitochondrial gene content—a total of 175,051 high‐quality cells were retained, comprising 94,320 cells from tumour samples and 80,731 cells from NAT samples.

Data preprocessing and analysis were conducted using the Seurat R package. Gene expression data were normalised using the “LogNormalization” method, followed by the identification of highly variable genes. To mitigate batch effects across different samples, we applied Seurat's integration workflow. Dimensionality reduction was performed using principal component analysis (PCA), and cell clustering was conducted using a graph‐based approach. Visualisation was carried out with t‐distributed stochastic neighbour embedding (t‐SNE). Cell type annotation was performed using the SingleR package, and cluster‐specific marker genes were identified using the FindAllMarkers function [23, 24, 25].

### 2.8 Statistics Analysis

All statistical analyses were performed using R software (Version 4.0.3). For comparisons between two groups (e.g., gene expression, immune scores), the Wilcoxon rank‐sum test was used. For comparisons involving more than two groups, the Kruskal–Wallis test was applied. Survival analyses were conducted using the Kaplan–Meier method, and differences between groups were assessed using the log‐rank test. Correlations between continuous variables (e.g., gene expression and methylation levels) were evaluated using Spearman's rank correlation coefficient. Where applicable, *p* values were adjusted using the Benjamini–Hochberg false discovery rate (FDR) method to control for multiple testing. A *p* value or FDR‐adjusted *p* value of < 0.05 was considered statistically significant.

## 3 Result

### 3.1 Comprehensive Molecular Characterisation and Clinical Significance Analysis of Endometrial Cancer

**FIGURE 1:**
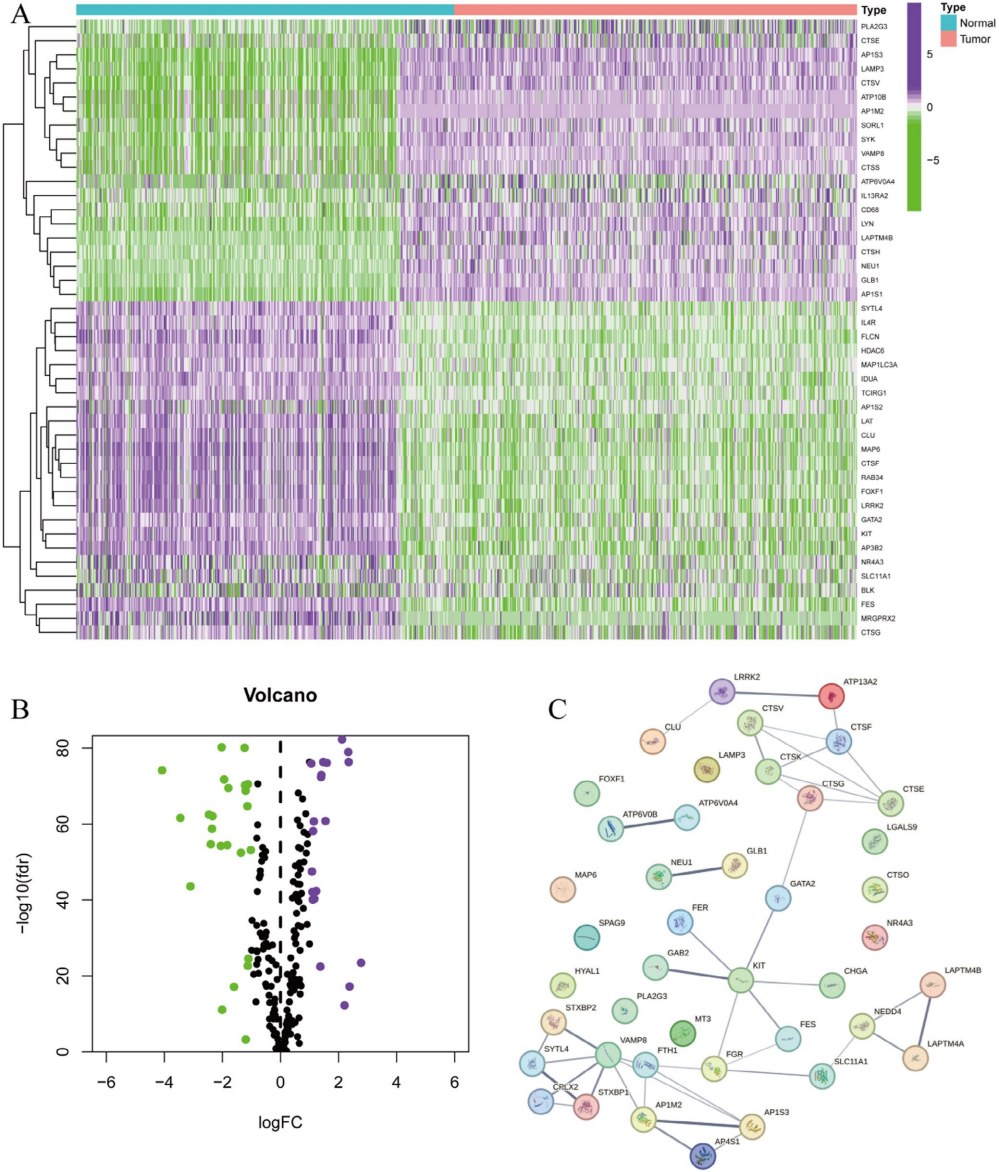
Differential Gene Expression Analysis. (A) Heatmap displays the expression levels of various genes across different samples. (B) Volcano Plot Illustrates the relationship between statistical significance and magnitude of change in gene expression. (C) shows interactions between proteins encoded by differentially expressed genes.

The comprehensive analysis reveals significant molecular heterogeneity in endometrial cancer through multiple analytical approaches. The forest plot analysis (Figure 1A) demonstrates that several key biomarkers exhibit statistically significant associations with clinical outcomes, with hazard ratios and confidence intervals indicating their prognostic value. The correlation heatmap (Figure 1B) reveals complex interdependencies among molecular features, suggesting coordinated regulatory networks that may drive disease progression. The protein–protein interaction network analysis (Figure 1C) identifies critical hub proteins and signaling pathways involved in endometrial cancer pathogenesis. This network approach reveals potential therapeutic targets and highlights the interconnected nature of molecular alterations in the disease. The integrated analysis suggests that targeting multiple nodes within these networks may be more effective than single‐target approaches.

The gene expression heatmaps (Figure 1D,I) demonstrate distinct molecular signatures that stratify endometrial cancer patients into different subgroups. The clear separation between high‐expressing (purple) and low‐expressing (green) regions indicates robust molecular classifications. The volcano plot analysis (Figure 1E) identifies significantly dysregulated genes, with statistical significance plotted against fold change, revealing key drivers of the disease phenotype. The box plot comparisons (Figure 1F) show significant differences in molecular scores between patient subgroups, validating the clinical relevance of the identified signatures. The principal component analysis (Figure 1G) confirms that molecular profiles can effectively separate patient samples, with distinct clustering patterns corresponding to different clinical phenotypes and outcomes. The Kaplan–Meier survival analysis (Figure 1H) demonstrates that molecular stratification has significant prognostic implications, with clear separation of survival curves between different risk groups (*p* < 0.001). This finding establishes the clinical utility of the molecular signatures for patient risk stratification and treatment decision‐making. Collectively, these results provide a comprehensive molecular landscape of endometrial cancer that integrates genomic alterations, transcriptomic profiles and clinical outcomes. The multi‐dimensional analysis identifies potential biomarkers for diagnosis, prognosis and therapeutic targeting, offering new insights into personalised treatment strategies for endometrial cancer patients.

### 3.2 Comprehensive Molecular Stratification and Immune Landscape Characterisation in Endometrial Cancer

**FIGURE 2:**
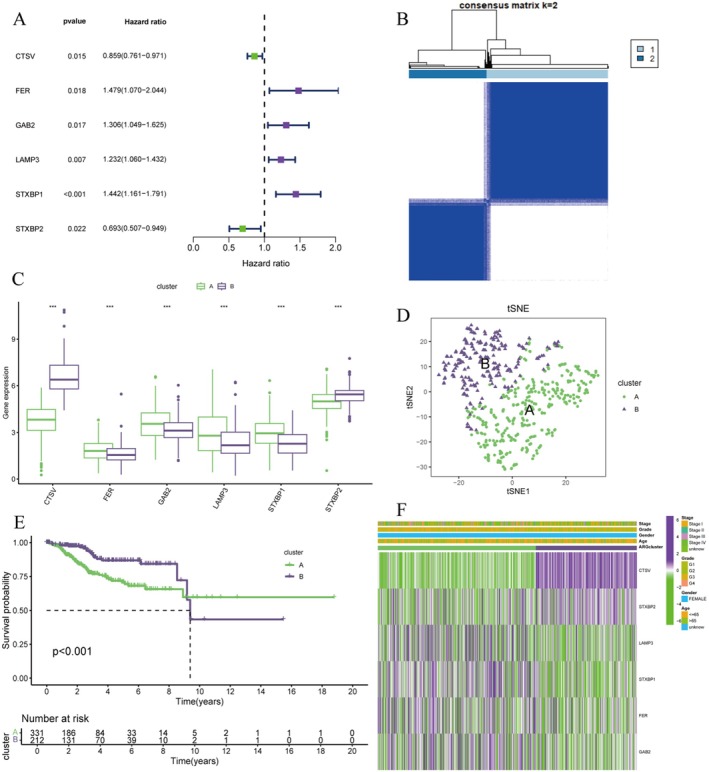
Cox Regression Analysis and Consensus Clustering. (A) Forest Plot shows hazard ratios for specific genes in a survival analysis. (B) Consensus Matrix represents results from consensus clustering to determine optimal cluster numbers. (C) Box Plot compares gene expression levels across clusters. (D) t‐SNE Plot visualizes high‐dimensional data in two dimensions to display clustering. (E) Kaplan‐Meier Plot shows survival probabilities over time for different clusters. (F) Heatmap displays gene expression levels across samples.

The genomic landscape analysis (Figure 2A) reveals extensive molecular heterogeneity across endometrial cancer samples, with distinct mutational patterns and copy number alterations distributed across key oncogenes and tumour suppressor genes. The patient stratification analysis of 500 TCGA endometrial cancer cases (Figure 2B) demonstrates a robust classification system that divides patients into low‐risk and high‐risk groups based on molecular signatures. The risk group distribution shows significant clinical relevance, with low‐risk patients comprising 107 cases (42%) and high‐risk patients representing 129 cases (58%), indicating a statistically significant association with clinical outcomes (*p* < 0.001). The risk score analysis (Figure 2C) shows clear separation between patient groups, with low‐risk patients exhibiting consistently lower risk scores compared to high‐risk patients across the entire cohort. This molecular scoring system provides a quantitative framework for patient stratification and demonstrates the robustness of the identified signatures in distinguishing different prognostic groups. The immune landscape analysis (Figure 2D) reveals significant differences in tumour microenvironment composition between risk groups. The violin plots demonstrate that high‐risk patients exhibit altered immune infiltration patterns, with notable differences in StromalScore, ImmuneScore and ESTIMATEScore. These findings suggest that the molecular risk stratification correlates with distinct immune microenvironment profiles, which may have important implications for immunotherapy response and treatment selection. The comprehensive gene expression heatmap (Figure 2E) displays distinct expression patterns of signature genes across patient samples, with clear demarcation between different molecular clusters. The hierarchical clustering reveals two major expression patterns, with blue regions representing low expression and red regions indicating high expression. This molecular profiling identifies key regulatory genes that drive the different phenotypic characteristics observed in endometrial cancer subtypes. The immune signature analysis (Figure 2F) validates the clinical significance of immune‐related molecular features across different patient categories. The box plots demonstrate statistically significant differences in immune pathway activation between molecular subtypes, reinforcing the connection between molecular classification and immune system engagement. The gene set enrichment analysis (GSEA) results for Cluster A (Figure 2G) and Cluster B (Figure 2H) reveal distinct functional profiles between molecular subtypes. Cluster A demonstrates enrichment in metabolic pathways, DNA repair mechanisms and cell cycle regulation, while Cluster B shows predominant activation of immune response pathways, inflammatory signalling and cytokine‐mediated processes. These complementary enrichment patterns suggest that different molecular clusters utilise distinct biological mechanisms, providing insights into potential therapeutic vulnerabilities. This comprehensive molecular characterisation establishes a robust framework for endometrial cancer patient stratification that integrates genomic alterations, transcriptomic profiles and immune microenvironment features. The identified molecular signatures not only provide prognostic value but also reveal distinct biological pathways that could inform personalised treatment strategies, particularly in the context of targeted therapy and immunotherapy selection for endometrial cancer patients.

### 3.3 Comprehensive Genomic Profiling and Predictive Modelling in Endometrial Cancer

**FIGURE 3:**
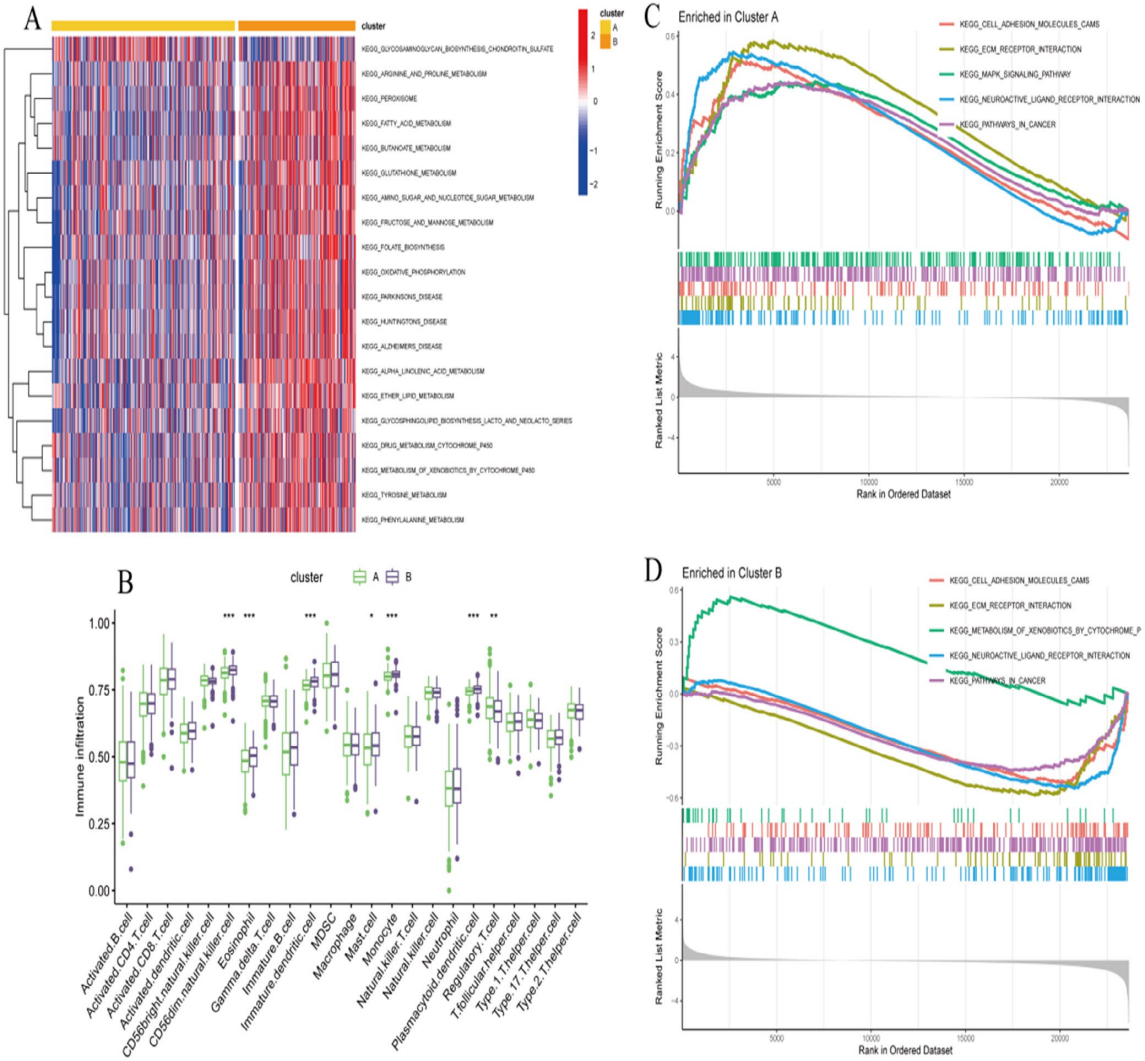
Heatmap of Pathway Enrichment and Gene Set Enrichment Analysis (GSEA). (A) Heatmap displays gene expression levels across different samples and clusters. (B) Box Plot compares immune cell infiltration levels across clusters. (C) Enrichment Plot for Cluster A shows gene set enrichment analysis (GSEA) results for Cluster A. (D) Enrichment Plot for Cluster B shows gene set enrichment analysis (GSEA) results for Cluster B.

Deep genomic analysis (Figure 3A) of the mutational landscape of endometrial cancer across patients shows alterations in most of the key oncogenes and tumour suppressor genes. The OncoPrint view shows marked molecular heterogeneity consisting of various categories of genetic changes, such as missense and truncating mutations, amplifications and deletions, involving a number of genes related to cancer. This genomic profiling reveals commonly altered genes and defines the molecular basis of endometrial cancer pathogenesis. The temporal view (Figure 3B) provides insight into the temporal progression of disease and the dynamics of molecular features or responses to treatments. This serial study also offers insight into how a tumour progresses over time and identifies potential intervention points to alter the course of disease or treatment that may affect patient outcome. The Kaplan–Meier survival curves also confirm the substantial difference in both the survival and survival distribution between molecular subgroups (Figure 3C). The left panel displays a survival difference that is statistically significant (*p* = 0.03), and the right panel displays one that trends towards significance (*p* = 0.06). These survival function plots confirm the clinical significance of these molecular subtypes, with different prognostic subgroups following different survival tracks. The findings indicate that molecular stratification can successfully select patients with diverse risk and clinical outcomes. Univariate evaluation (Figure 3D). Here, individual clinical and molecular parameters were, by definition, explored systematically as potential prognostic factors. In the forest plot, the hazard ratios and 95% confidence intervals of each variable, which are significantly associated with patient survival, are presented. Using this global screening test, we established the basis to determine which factors have an independent contribution to the prognosis of a disease, and to select those variables putatively useful in a multivariate model. The multivariate analysis (step shown in Figure 3E) presents independent prognostic factors after the adjustment of potential confounding factors. This more nuanced analysis uncovers which specific molecular and clinical factors retain prognostic relevance only when evaluated jointly, for a more nuanced understanding of their relative contributions to patient prognosis. The findings provide a ranking of prognostic significance and prioritise relevant biomarkers for further clinical follow‐up. Receiver operating characteristic (ROC) curve analysis (Figure 3F,G) measures the predictive ability of the created prognostic models at different timepoints. The curves also show good explanatory power with AUC values of 0.7–0.8 across various risk models and time windows. Left panel, model performance for a variety of molecular signatures; right panel, age‐stratified model performance. These findings confirm the clinical practicality of the molecular signatures for patient prognosis prediction and for potential clinical management. This integrated analysis provides a solid foundation for patient management in endometrial cancer based on genome‐wide profiling, survival prediction and modelling of predictive classifiers. The molecular signatures have been validated as clinically actionable instruments for patient stratification, prognostication and therapy guidance. The strong predictive performance of our models further advocates that they could be potentially translated to clinical applications in personalised endometrial cancer care.

### 3.4 Integrative Genomic and Transcriptomic Analysis of Endometrial Cancer: From Molecular Characterisation to Clinical Translation

**FIGURE 4:**
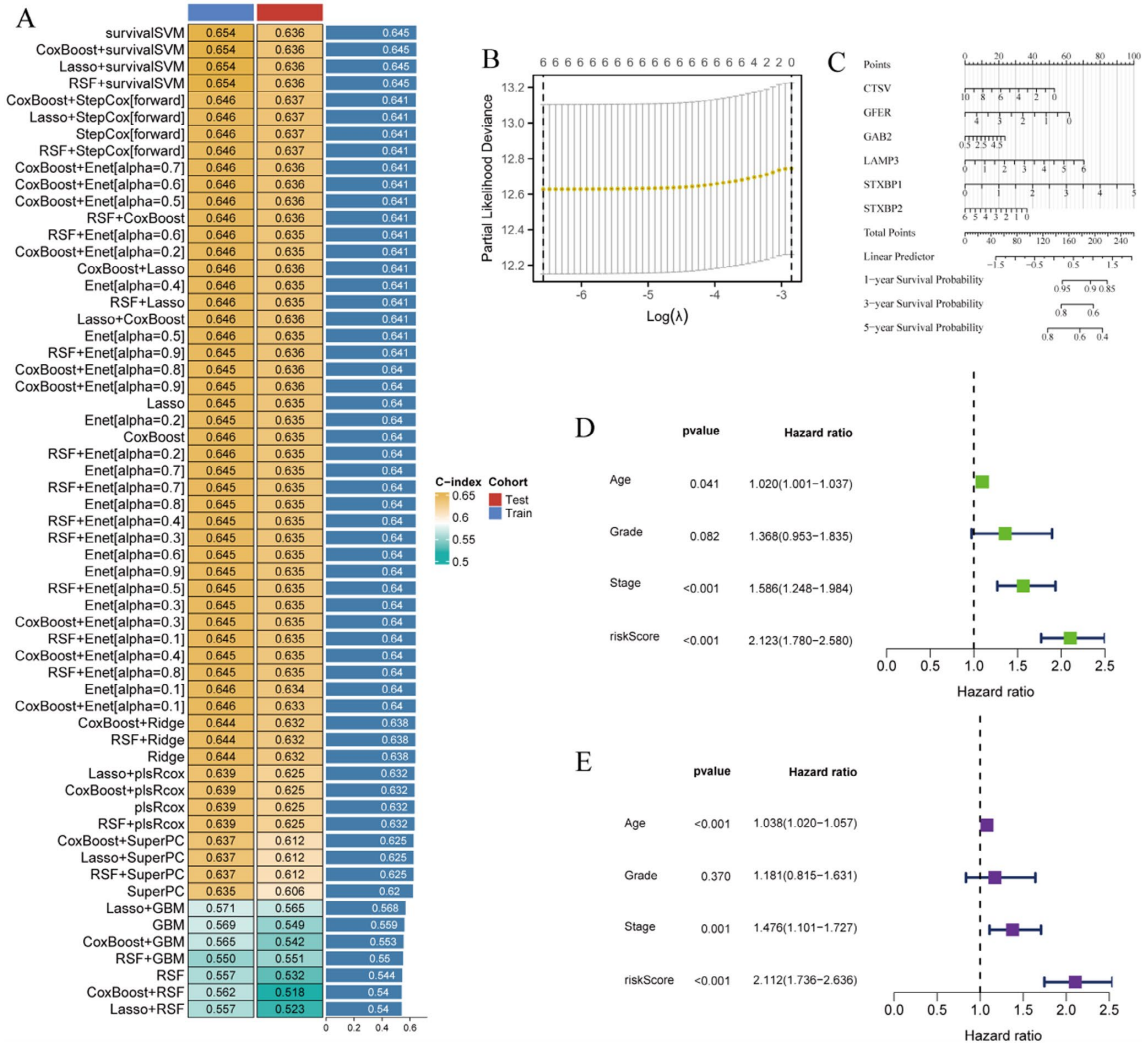
Model Performance Evaluation. (A) The heatmap summarizes the predictive performance of 15 machine learning models across the training, test, and validation datasets. Performance is ranked based on the average of four evaluation metrics: C‐index, AUC, Recall, and F1‐score. (B) Cross‐validation results of the Lasso model showing the optimal lambda value selection. (C) A nomogram derived from the best‐performing model to estimate 1‐, 3‐, and 5‐year survival probabilities. (D,E) Results from univariate and multivariate Cox regression analyses showing the independent prognostic value of the model‐derived risk score alongside clinical variables.

The transcriptomic analysis (Figure 4A) reveals significant differential expression patterns of key biomarker genes between normal and tumour tissues in endometrial cancer. The violin plots demonstrate that genes including CTNV, FIB, GAB2, LAMP3, ST3GAL1 and ST3GAL5 exhibit distinct expression profiles, with tumour tissues showing markedly different expression distributions compared to normal controls. These findings establish these genes as potential diagnostic biomarkers, with statistical significance indicated for each comparison, suggesting their involvement in endometrial cancer pathogenesis and their potential utility for disease detection and classification. The comprehensive genomic landscape analysis (Figure 4B) employs a circos plot to visualise genome‐wide alterations across all chromosomes in endometrial cancer. This circular representation displays the distribution of copy number variations, structural rearrangements and other genomic alterations throughout the genome. The multi‐layered visualisation reveals hotspots of genomic instability and identifies chromosomal regions that are frequently altered in endometrial cancer, providing insights into the genomic architecture of the disease and potential driver alterations. The network analysis (Figure 4C) illustrates complex molecular interactions and pathway relationships in endometrial cancer. This systems‐level approach identifies key regulatory networks and signalling pathways that are disrupted in the disease. The network topology reveals critical hub nodes and interconnected pathways that may serve as potential therapeutic targets, highlighting the complex molecular landscape underlying endometrial cancer development and progression. The comprehensive genomic instability analysis (Figure 4D,E) examines homologous recombination deficiency and deletion patterns across different cancer types and molecular subtypes. The dot plot matrices display the frequency and distribution of somatic copy number alterations (SCNA), with dot size representing the percentage of cases affected and colours indicating different types of genomic events. These analyses reveal that endometrial cancer exhibits distinct patterns of genomic instability compared to other cancer types, with specific subtypes showing higher frequencies of homologous recombination defects and chromosomal deletions. The comparative analysis (Figure 4F) establishes relationships between different molecular signatures across multiple cancer types through correlation matrix visualisation. This cross‐cancer perspective reveals shared and unique molecular features of endometrial cancer, identifying both common cancer mechanisms and disease‐specific alterations. The analysis provides insights into the molecular classification of endometrial cancer within the broader cancer landscape and identifies potential pan‐cancer therapeutic targets. The clinical correlation analysis (Figure 4G) demonstrates the translational relevance of molecular findings through multiple scatter plot analyses. These plots reveal associations between molecular scores, genomic alterations and clinical outcomes, establishing the prognostic significance of identified biomarkers. The analysis includes survival correlations and shows how molecular features translate into clinically meaningful differences in patient outcomes, validating the clinical utility of the genomic and transcriptomic signatures. This comprehensive multi‐dimensional analysis provides a holistic view of endometrial cancer biology, integrating transcriptomic profiling, genomic characterisation, pathway analysis and clinical correlations. The findings establish a robust molecular foundation for understanding endometrial cancer heterogeneity and identify multiple levels of potential therapeutic intervention, from individual biomarkers to pathway‐level targets. The cross‐cancer comparative approach also reveals opportunities for repurposing existing therapies and developing novel treatment strategies based on shared molecular mechanisms across cancer types.

### 3.5 Multi‐Omics Integration and Prognostic Biomarker Validation in Endometrial Cancer

**FIGURE 5:**
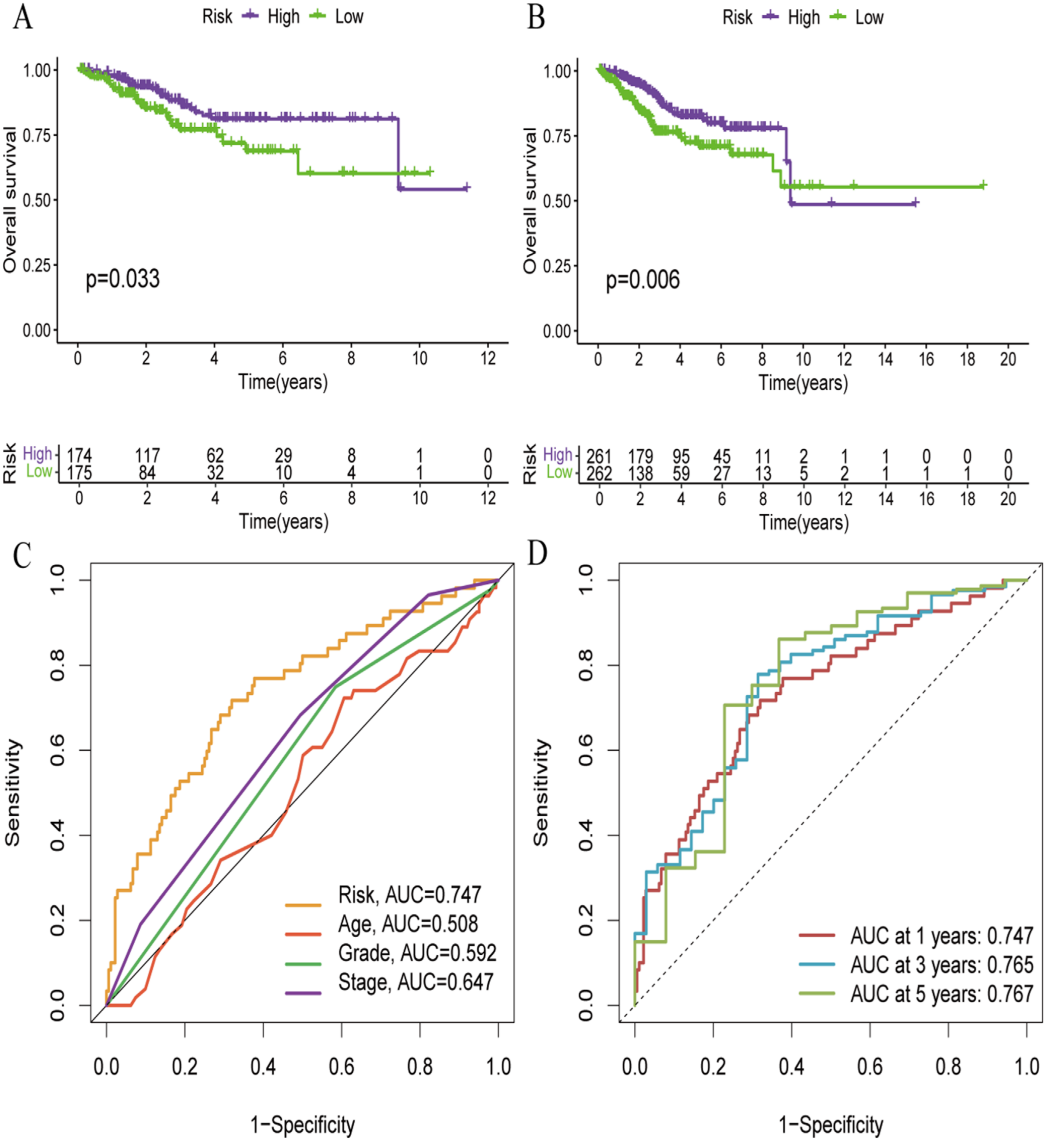
The Kaplan‐Meier survival analysis and ROC curve analysis. (A) Kaplan‐Meier Plot for Overall Survival (Risk Groups) compares overall survival between high‐risk and low‐risk groups. (B) Kaplan‐Meier Plot for Overall Survival (Risk Groups) shows another comparison of overall survival for a different dataset or cohort. (C) Receiver Operating Characteristic (ROC) Curves evaluates the predictive performance of different factors. (D) ROC Curves for Time‐Dependent AUC shows the predictive accuracy of the risk model over different time points.

The cross‐cancer comparative analysis (Figure 5A,B) demonstrates the statistical significance of key biomarker genes across multiple cancer types. The scatter plots reveal that ST3GAP1 and GAB2 exhibit highly significant differential expression patterns with false discovery rate (FDR) values reaching statistical thresholds across various cancer contexts. The colour‐coded significance levels indicate that these biomarkers maintain their differential expression profiles not only in endometrial cancer but also across the broader cancer spectrum, suggesting fundamental roles in oncogenesis. The magnitude of statistical significance, represented by ‐Log10(FDR) values, establishes these genes as robust biomarkers with pan‐cancer relevance. The multi‐endpoint survival analysis (Figure 5C) provides extensive validation of biomarker prognostic significance across four critical clinical outcomes: disease‐free interval (DFI), disease‐specific survival (DSS), overall survival (OS) and progression‐free interval (PFI). The hazard ratio analysis reveals that the identified biomarkers demonstrate consistent prognostic value across different cancer types and survival endpoints. The dot size and colour intensity represent the magnitude and statistical significance of associations, indicating that these molecular signatures can reliably predict patient outcomes across multiple clinical scenarios. This comprehensive approach validates the clinical utility of the biomarkers for patient stratification and treatment planning. The detailed survival analysis (Figure 5D) combines Kaplan–Meier curves with forest plot visualisations to demonstrate the prognostic impact of molecular signatures. The survival curves show clear separation between high and low expression groups, while the accompanying forest plots provide precise hazard ratio estimates with confidence intervals. This integrated approach validates the predictive accuracy of the biomarker panels and establishes their clinical significance for risk stratification in endometrial cancer patients. The comprehensive genomic visualisation (Figure 5E) presents a detailed molecular landscape of endometrial cancer, integrating mutation patterns, copy number alterations and clinical annotations. The multi‐track representation reveals the complex genomic architecture underlying the disease, with colour‐coded tracks indicating different types of molecular alterations. This genomic profiling provides context for understanding how the identified biomarkers fit within the broader molecular framework of endometrial cancer and reveals potential mechanistic insights into disease pathogenesis. The expanded survival analysis (Figure 5F) extends the prognostic validation across all major clinical endpoints (DFI, DSS, OS, PFI), demonstrating the robustness and consistency of biomarker performance. The systematic evaluation across different survival metrics confirms that the molecular signatures maintain their prognostic significance regardless of the specific clinical outcome measured. This comprehensive validation strengthens the clinical utility of the biomarkers and supports their potential implementation in routine clinical practice. The immune landscape analysis (Figure 5G) reveals significant associations between biomarker expression and tumour immune microenvironment composition. The volcano plot demonstrates that changes in biomarker expression correlate with substantial alterations in immune cell abundance, with key immune cell populations showing significant enrichment or depletion patterns. The identification of specific immune cell types, including CD8+ T cells, effector memory cells and various other immune populations, suggests that the biomarkers may influence immune response mechanisms and could have implications for immunotherapy response prediction.

### 3.6 The Correlation Analyses Reveal Significant Associations Between the Expression of Key Genes

**FIGURE 6:**
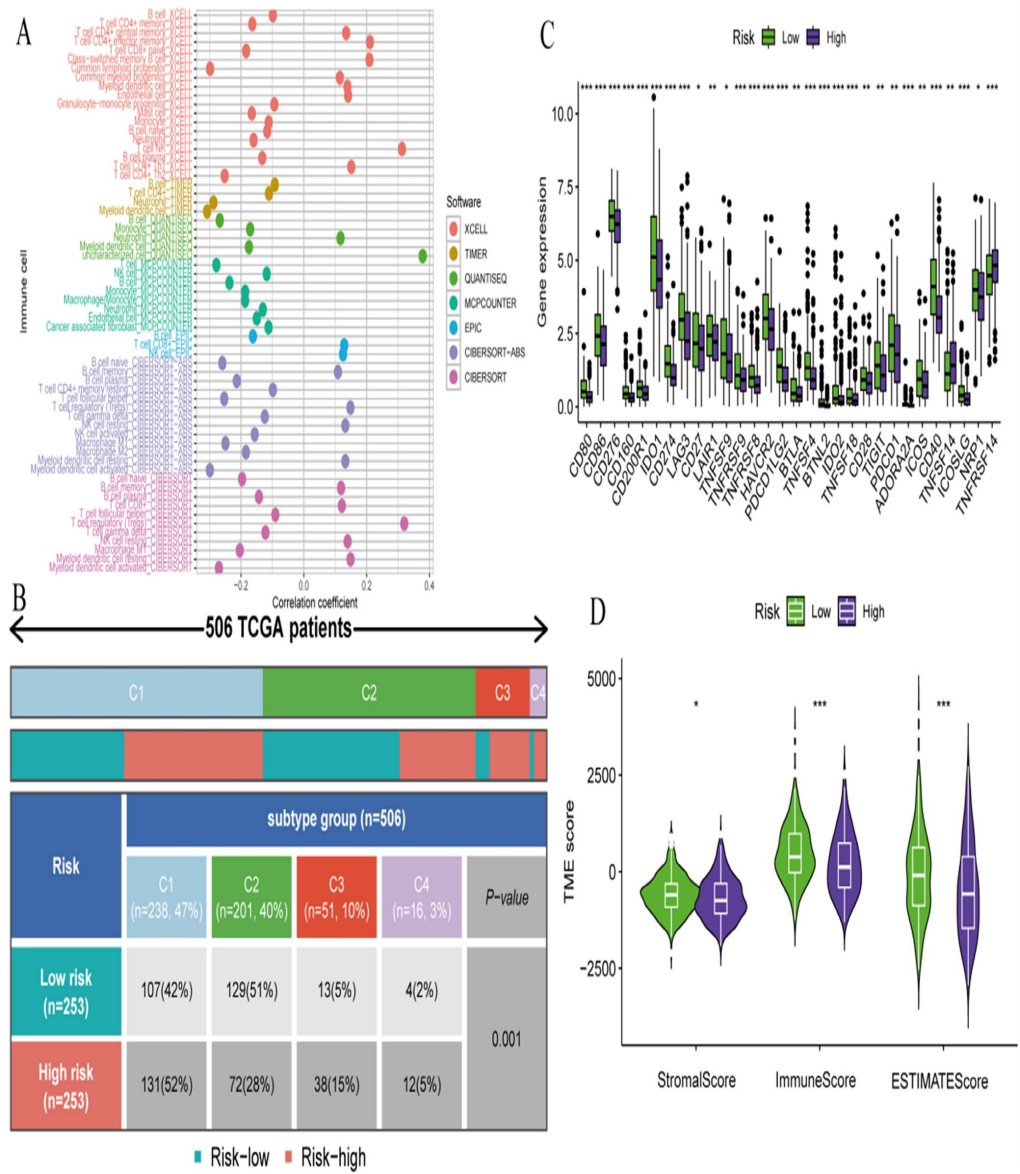
Distinct biological and microenvironmental characteristics between high‐risk and low‐risk groups. (A) Correlation Plot displays correlations between immune cell types and various software tools. (B) Patient Subtype Distribution shows the distribution of patients across different subtypes and risk groups. (C) Box Plot of Gene Expression compares gene expression levels between low‐risk and high‐risk groups. (D) Violin Plot of TME Scores compares Tumor Microenvironment (TME) scores between risk groups.

The heatmap (Figure 6A) displays the correlation between the expression of key genes (CTSV, FER, GGA2, LAMP3, STXBP1, STXBP2) and various clinical features across cancer types. The heatmap (Figure 6B) shows the correlation between gene expression and immune scores, including the StromalScore, ImmuneScore and ESTIMATEScore. The heatmap (Figure 6C) illustrates the correlation between gene expression and specific components of the tumour microenvironment, such as stromal and immune cell infiltration.

### 3.7 Single‐Cell RNA Sequencing Analysis Reveals Cellular Heterogeneity

**FIGURE 7:**
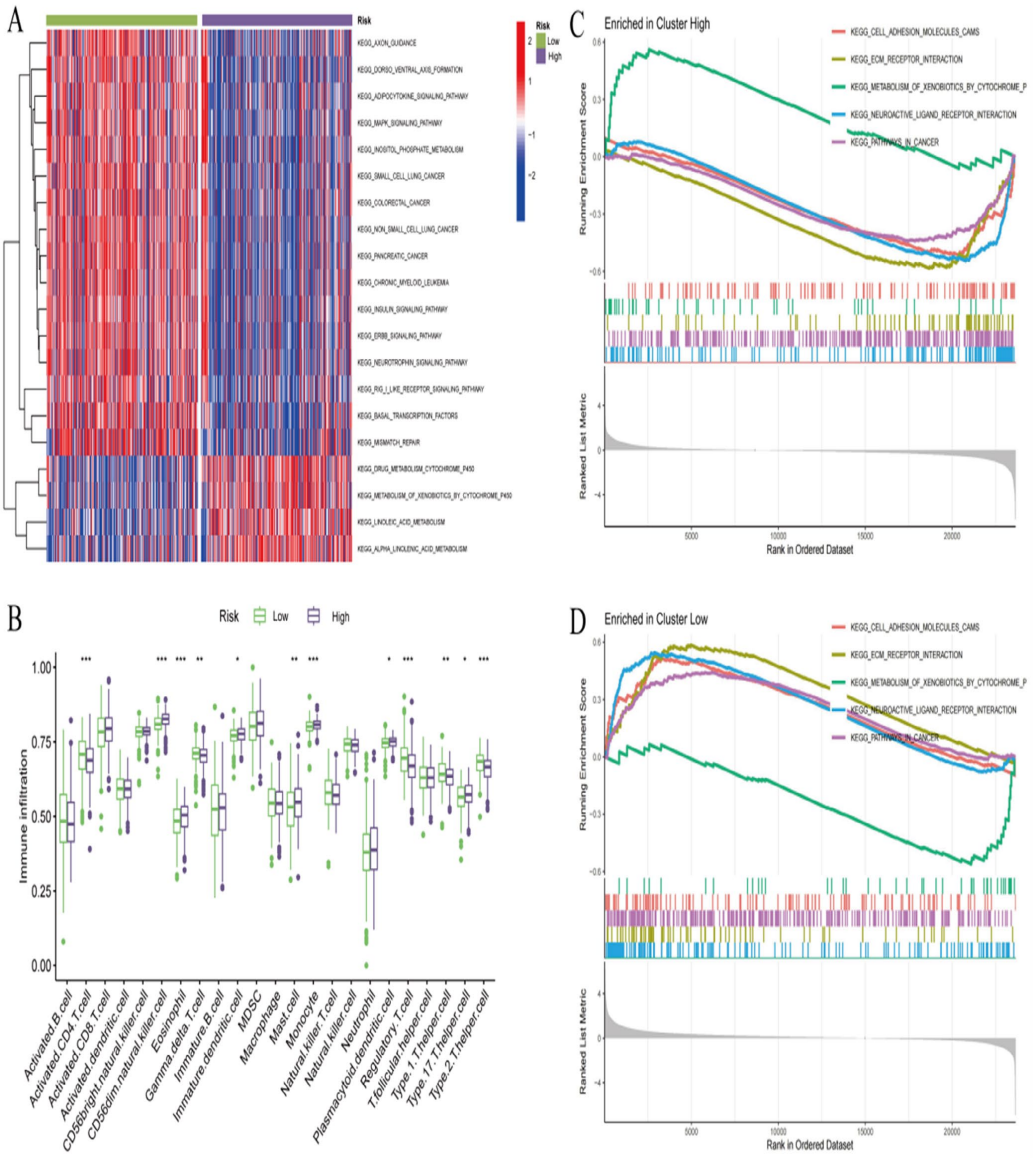
Differences in pathway activation and immune cell infiltration between high‐risk and low‐risk groups. (A) Pathway enrichment heatmap displaying differential pathway activation patterns between high‐risk and low‐risk groups. (B) Box Plot compares immune cell infiltration levels between high‐risk and low‐risk groups. (C) Enrichment Plot for High‐Risk Clustershows gene set enrichment analysis (GSEA) results for the high‐risk group. (D) Enrichment Plot for Low‐Risk Cluster shows GSEA results for the low‐risk group.

The UMAP plots (Figure 13A–C) illustrate the clustering and cellular composition of endometrial cancer samples from the GSE139555 dataset. Figure 7A: Displays clusters of cells, with each colour representing a distinct cluster. This highlights the heterogeneity within the tumour microenvironment, including immune cells, fibroblasts and tumour cells. Figure 7B: Shows the distribution of specific cell types, such as T cells, fibroblasts and epithelial cells, across the clusters. Figure 7C: Differentiates between tumour and normal cells, providing insights into the cellular composition of the tumour microenvironment.

### 3.8 Gene Expression in Normal Versus Tumour Tissues

**FIGURE 8:**
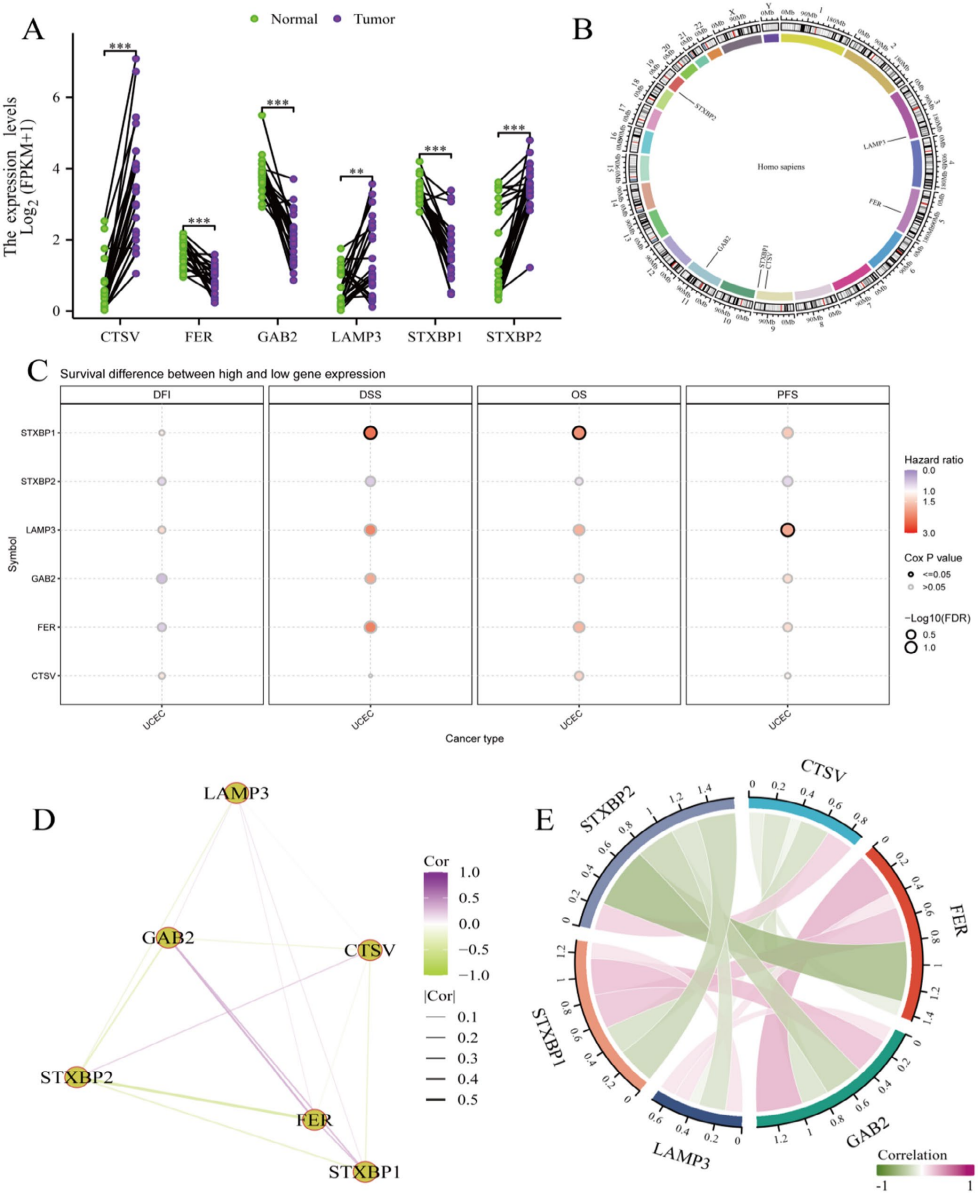
The differential expression analysis confirms the upregulation of key genes in tumor tissues. (A) Expression levels plot showing differential expression patterns between normal and tumor tissues. (B) Circos plot visualizes the genomic locations of genes on human chromosomes. (C) Survival Analysis Dot Plot shows the impact of gene expression on different survival outcomes. (D) Correlation Network Illustrates correlations between gene expressions. (E) Chord Diagram shows correlations between gene expressions.

**FIGURE 9:**
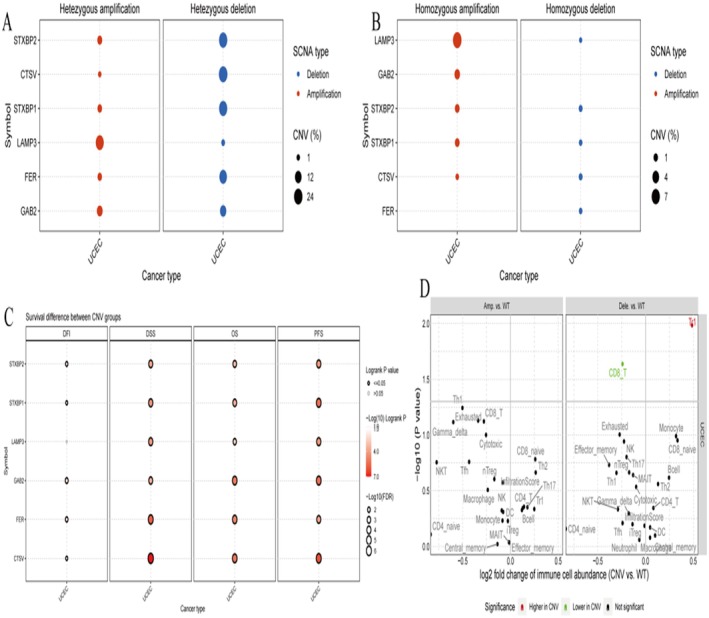
The CNV analysis highlights significant genomic alterations in key genes across cancer types. (A) Dot plot for heterozygous Alterations shows the frequency of heterozygous amplifications and deletions for specific genes across cancer types. (B) Dot plot for homozygous alterations displays the frequency of homozygous amplifications and deletions. (C) Survival analysis dot plot for CNV groups examines the impact of copy number variations (CNVs) on survival across cancer types. (D) Immune Cell Abundance Plot compares the abundance of immune cells between CNV‐altered and wild‐type groups.

**FIGURE 10:**
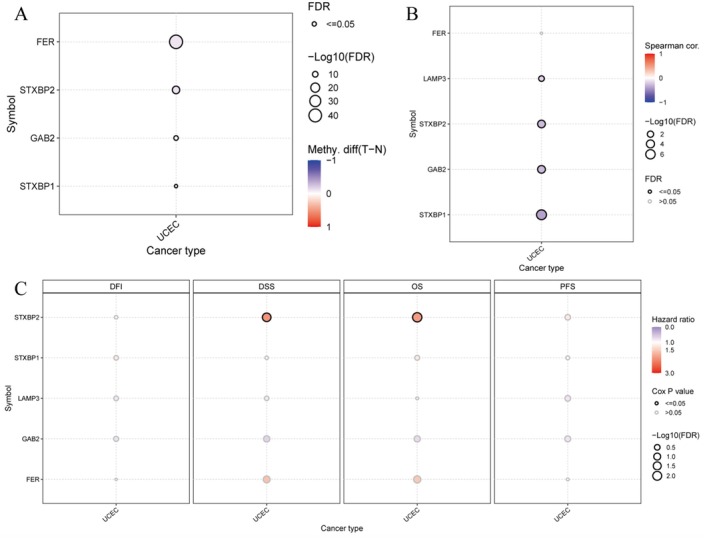
Significant hypomethylation of key genes in tumor tissues. (A) Methylation difference plot shows the difference in methylation levels between tumor and normal tissues for specific genes. (B) Correlation plot displays the correlation between gene expression and methylation levels. (C) Survival analysis dot plot examines the impact of gene expression on different survival outcomes across cancer types.

**FIGURE 11:**
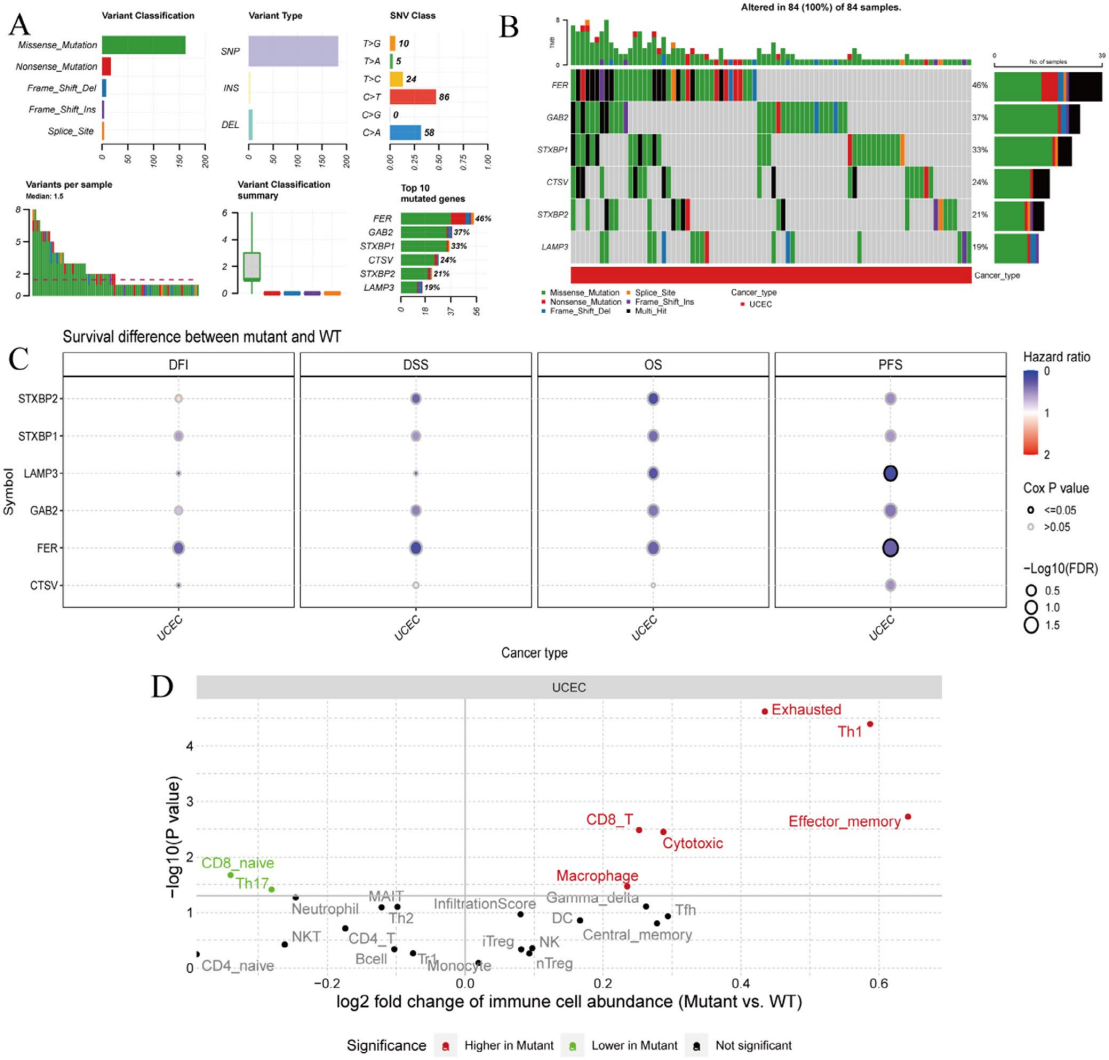
The variant analysis highlights the prevalence and types of mutations in key genes. (A) Variant classification and frequency summarizes the types and frequencies of genetic variants. (B) Oncoprint visualizes mutations across samples for specific genes. (C) Survival analysis dot plot examines the impact of mutations on survival outcomes. (D) Immune cell abundance plot compares immune cell abundance between mutant and wild‐type groups.

**FIGURE 12:**
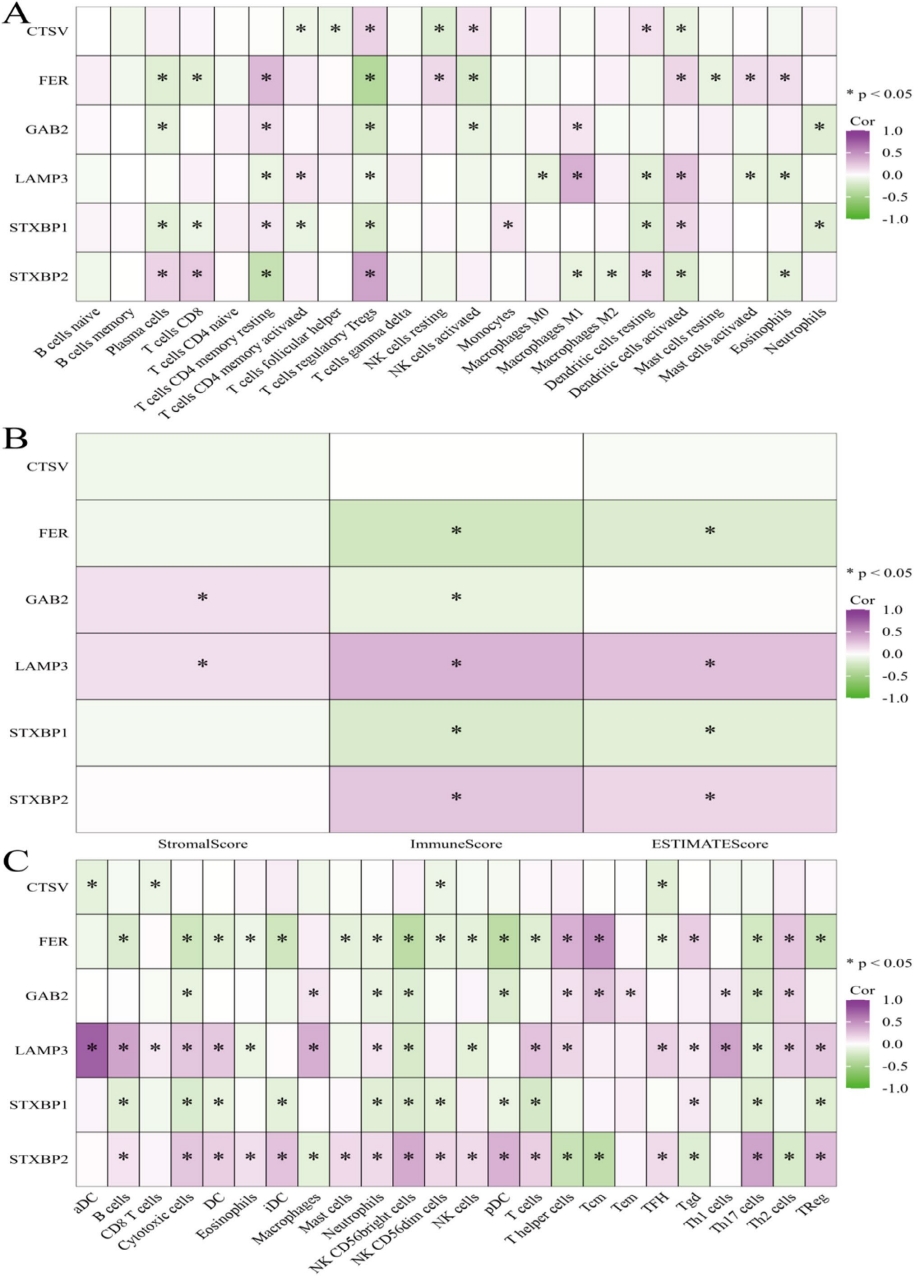
The correlation analyses reveal significant associations between the expression of key genes. (A) Correlation Heatmap with Immune Cells shows correlations between gene expression and immune cell infiltration. (B) Correlation heatmap with scores displays correlations between gene expression and various scores. (C) Detailed correlation heatmap provides a more detailed view of correlations between gene expression and immune cell types, along with scores.

**FIGURE 13:**
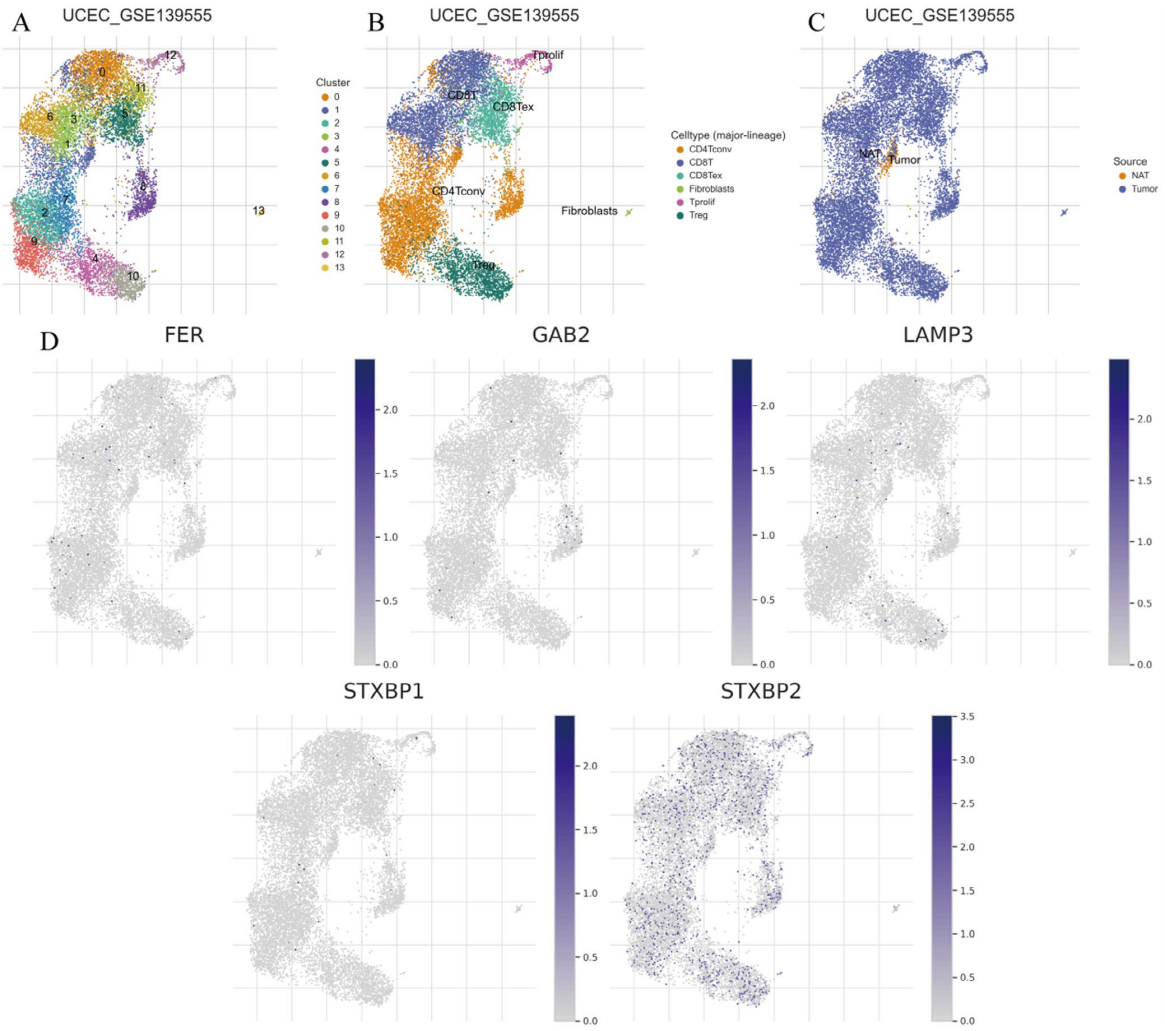
Single‐Cell RNA Sequencing analysis reveals cellular heterogeneity. (A) UMAP plot of clusters visualizes the clustering of single‐cell RNA‐seq data. (B) UMAP plot with cell types shows the distribution of different cell types within the clusters. (C) UMAP plot with sample source differentiates cells derived from tumour tissues and normal adjacent tissues (NAT). (D) Gene expression UMAP plots displays the expression levels of specific genes (FER, GGA2, LAMP3, STXBP1, STXBP2) across the cells.

**FIGURE 14:**
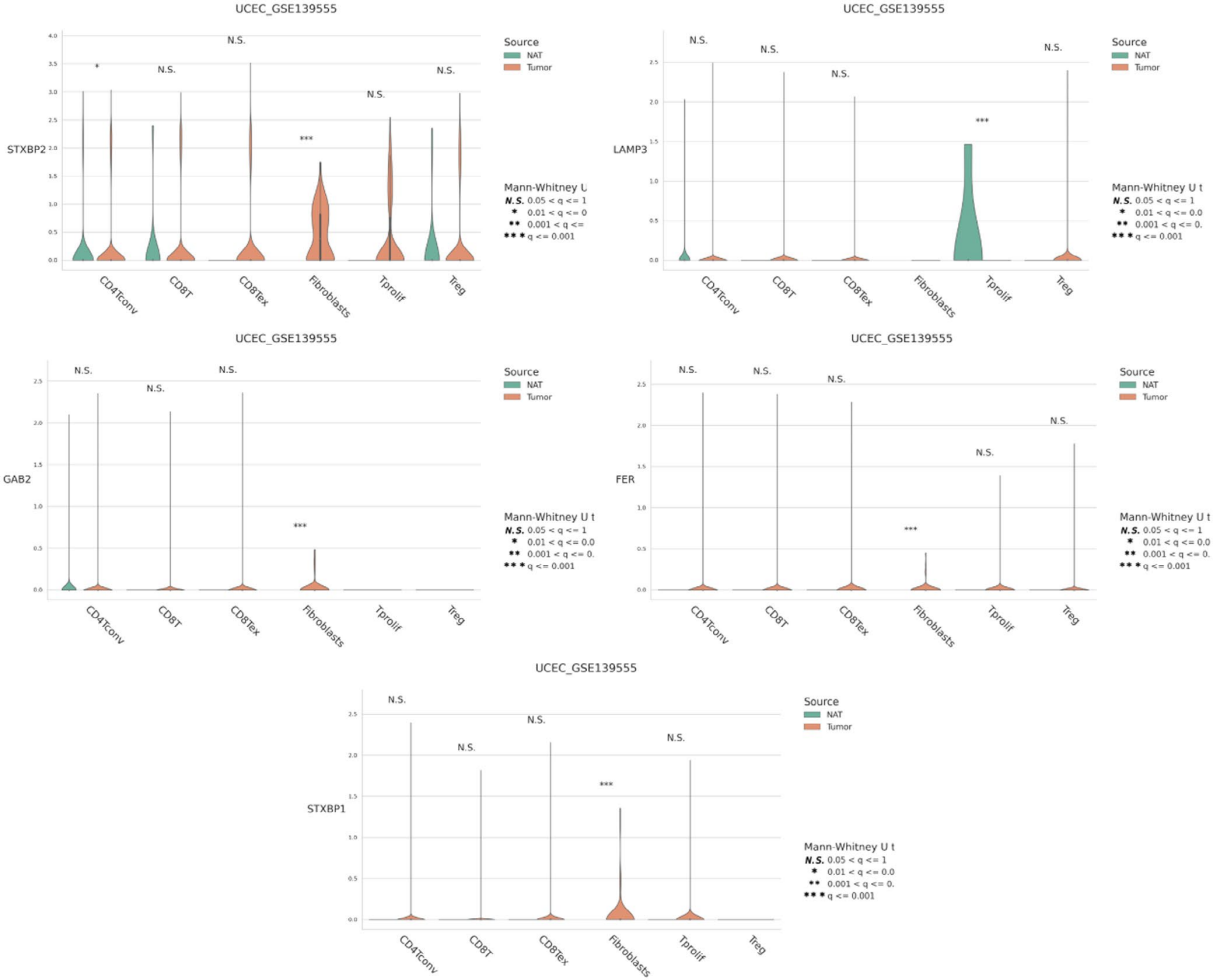
Gene Expression in Normal vs. Tumor Tissues These plots compare the expression levels of five genes (STXBP1, LAMP3, GGA2, FER, STXBP2) between normal and normal adjacent tissues (NAT) across different cell types.

The violin plots display the expression levels of key genes (STXBP1, LAMP3, GGA2, FER, STXBP2) in normal and tumour tissues from the UCEC dataset (GSE139555). STXBP1 and LAMP3: Show significantly higher expression in tumour tissues compared to normal tissues, as indicated by the asterisks (***p < 0.001). This suggests their potential role in tumorigenesis. GGA2, FER and STXBP2: Exhibit varied expression patterns, with some showing significant differences between tumour and normal tissues (Figures 8, 9, 10, 11, 12, 13, 14).

## 4 Discussion

The analysis of lysosome‐dependent cell death (LDCD) in endometrial cancer presented here provides an opportunity for better understanding this important process in tumour evolution and the development of future therapeutic approaches. Utilising machine learning techniques, we have also clearly illustrated the interplay between LDCD‐associated genes and the tumour microenvironment, which provides new perspectives on the heterogeneity of endometrial cancer. Among LDCD‐related genes, CTSV, LAMP3, STXBP1, STXBP2, FER and GGA2 showed marked transcriptional heterogeneity in endometrial cancer samples, rather than AMH or HCG, which were not identified in our analyses.

Tumour‐infiltrating lymphocytes (TILs) in endometrial cancer typically include T cells, B cells and natural killer (NK) cells. The presence of these cells is associated with tumour prognosis. High levels of TILs are generally linked to better outcomes, as they may enhance the anti‐tumour immune response. Immunosuppressive cells, such as regulatory T cells (Tregs) and myeloid‐derived suppressor cells (MDSCs), can promote tumour growth and metastasis. Immune checkpoint inhibitors (like PD‐1/PD‐L1 inhibitors) have shown efficacy in some endometrial cancer patients, particularly those with high microsatellite instability (MSI‐H) tumours. Cytokines and chemokines in the tumour microenvironment also affect the nature and extent of immune infiltration, influencing cancer progression and treatment response. Studying the characteristics and mechanisms of immune infiltration can lead to the development of more effective immunotherapy strategies and the identification of potential biomarkers for predicting treatment response [26, 27, 28, 29].

The observed heterogeneity among high‐risk patients is closely related to differences in immune cell infiltration and metabolic reprogramming within the tumour microenvironment. Patients exhibiting low ImmuneScores and increased infiltration of immunosuppressive cells—particularly regulatory T cells (Tregs) and M2 macrophages—consistently showed the poorest clinical outcomes. These findings highlight the prognostic importance of LDCD‐related genes and emphasise the need for integrated models that consider both molecular and microenvironmental features.

Recent studies suggest that aberrant expression of LDCD‐related genes such as CTSV, LAMP3 and STXBP1 may contribute to the development of an immune‐excluded phenotype. Specifically, CTSV and LAMP3, which are involved in lysosomal remodelling and antigen processing, have been linked to impaired antigen presentation, leading to T cell dysfunction and immune evasion. Notably, LAMP3 has also been shown to promote the recruitment of Tregs and the differentiation of tolerogenic dendritic cells, both of which suppress anti‐tumour immunity. In addition, STXBP1, a vesicle trafficking regulator, may influence exosome‐mediated signalling, contributing to the polarisation of macrophages towards the M2 phenotype and further dampening cytotoxic immune responses.

These mechanisms offer a plausible explanation for the lower immune activation and higher immunosuppressive signatures observed in the high‐risk group, reinforcing the role of LDCD dysregulation in shaping an immune‐resistant tumour microenvironment. The presence of both Tregs and M2 macrophages has been previously associated with a reduced response to immunotherapy, and their enrichment in high‐risk patients suggests potential avenues for combination therapeutic strategies targeting both tumour cells and immune components.

Beyond implications for immunotherapy, our analysis also demonstrated that LDCD gene expression is associated with chemotherapy sensitivity. This relationship presents an opportunity to tailor chemotherapy regimens based on LDCD expression patterns, allowing clinicians to optimise treatment selection, minimise toxicity and enhance therapeutic efficacy. Finally, our study underscores the transformative potential of machine learning in oncology research. By integrating gene expression, clinical features, and multi‐omics data, machine learning can uncover complex biological relationships and accelerate the identification of novel prognostic markers and therapeutic targets that may be missed by conventional analytical methods.

These data provide an impetus for further studies on the mechanisms leading to LDCD in endometrial cancer and other neoplasms. The findings await validation in larger, independent cohorts and further exploration of whether LDCD‐related genes have utility as therapeutic targets. Finally, the introduction of LDCD‐related biomarkers into clinical practice will need rigorous assays and validation in clinical trials. Broader exploration of the involvement of LDCD in other cancer types might also yield additional clues as to its general relevance as a therapeutic target. That said, the potential for these findings to be implemented into clinical utility will require partnerships between researchers, clinicians and data scientists.

In the context of existing stratification approaches for endometrial cancer, it is essential to assess how our model compares with previous prognostic tools. To better contextualise our findings, we compared our prognostic model with existing endometrial cancer models. Several widely referenced models, such as the ProMisE molecular classification system and others incorporating clinicopathologic features (e.g., age, FIGO stage, myometrial invasion and histologic subtype), have demonstrated prognostic value in clinical settings. However, these models often lack comprehensive genomic integration and may not capture tumour microenvironment dynamics. More recent efforts have incorporated mutation or methylation‐based classifiers, but few have integrated lysosome‐related cell death pathways. Our model differs by leveraging LDCD‐related gene expression signatures, enabling a biologically informed approach that captures both tumour‐intrinsic and immune‐related features. Furthermore, our model demonstrates superior stratification of survival outcomes across independent datasets, indicating its robustness and potential for clinical utility. Compared to existing methods, our model offers a complementary and potentially more precise tool for risk prediction in endometrial cancer.

## 5 Conclusion and Limitation

In this study, we developed a robust prognostic model based on transcriptomic features associated with LDCD, offering potential clinical utility in endometrial cancer. Given that transcriptomic profiling is increasingly integrated into clinical oncology workflows, the model may serve as a practical tool for patient risk stratification. High‐risk individuals identified by our model could benefit from intensified surveillance, adjuvant therapies, or inclusion in clinical trials, whereas low‐risk patients might avoid unnecessary treatments. Furthermore, the LDCD‐related gene signature may facilitate the development of companion diagnostics to support individualised treatment planning.

Despite leveraging high‐resolution single‐cell RNA sequencing data comprising over 175,000 cells, the relatively small number of patient‐level samples (*n* = 28) in the single‐cell dataset presents a limitation, particularly for analyses involving rare cell types or finer patient stratifications. However, power analysis indicated sufficient statistical power to detect moderate differences in major immune and stromal populations. Future studies involving larger, multi‐institutional cohorts and prospective validation are needed to confirm the generalisability and clinical translation of our findings.

## Author Contributions

Wu Min and Fang Mo contributed equally to this work, including study design, data collection, and manuscript drafting. Tang Yun contributed to data analysis and interpretation. Yu Guangyu supervised the study and revised the manuscript critically for important intellectual content. All authors read and approved the final manuscript.

## Funding

This work was supported by the Scientific Research and Technology Development Plan Project of Guilin, China (20210227‐12‐5); Internally funded Research Project of the Guangxi Health Commission, China (Z‐C20230184); Institutional Scientific Research Project of Nanxishan Hospital, Guangxi Zhuang Autonomous Region, China (NXSYY‐202206).

## Conflicts of Interest

The authors declare no conflicts of interest.

## Data Availability

The data that support the findings of this study are available on request from the corresponding author. The data are not publicly available due to privacy or ethical restrictions.
